# Genetic analysis of the early bud flush trait of tea plants (*Camellia sinensis*) in the cultivar ‘Emei Wenchun’ and its open-pollinated offspring

**DOI:** 10.1093/hr/uhac086

**Published:** 2022-04-21

**Authors:** Liqiang Tan, Dong Cui, Liubin Wang, Qinling Liu, Dongyang Zhang, Xiaoli Hu, Yidan Fu, Shengxiang Chen, Yao Zou, Wei Chen, Weiqi Wen, Xuemei Yang, Yang Yang, Pinwu Li, Qian Tang

**Affiliations:** 1 College of Horticulture, Sichuan Agricultural University, Chengdu 611130, Sichuan, China; 2 Tea Refining and Innovation Key Laboratory of Sichuan Province, Chengdu 611130, Sichuan, China; 3 Mingshan Tea Plant Breeding and Reproduce Farm of Sichuan Province, Yaan 625101, Sichuan, China; 4 Sichuan Yizhichun Tea Industry Co., Ltd,, Leshan 614503, Sichuan, China

## Abstract

The timing of bud flush (TBF) in the spring is one of the most important agronomic traits of tea plants (*Camellia sinensis*). In this study, we designed an open-pollination breeding program using ‘Emei Wenchun’ (EW, a clonal tea cultivar with extra-early TBF) as a female parent. A half-sib population (*n* = 388) was selected for genotyping using specific-locus amplified fragment sequencing. The results enabled the identification of paternity for 294 (75.8%) of the offspring, including 11 (2.8%) from EW selfing and 217 (55.9%) assigned to a common father, ‘Chuanmu 217’ (CM). The putative EW × CM full-sib population was used to construct a linkage map. The map has 4244 markers distributed in 15 linkage groups, with an average marker distance of 0.34 cM. A high degree of collinearity between the linkage map and physical map was observed. Sprouting index, a trait closely related to TBF, was recorded for the offspring population in 2020 and 2021. The trait had moderate variation, with coefficients of variation of 18.5 and 17.6% in 2020 and 2021, respectively. Quantitative trait locus (QTL) mapping that was performed using the linkage map identified two major QTLs and three minor QTLs related to the sprouting index. These QTLs are distributed on Chr3, Chr4, Chr5, Chr9, and Chr14 of the reference genome. A total of 1960 predicted genes were found within the confidence intervals of QTLs, and 22 key candidate genes that underlie these QTLs were preliminarily screened. These results are important for breeding and understanding the genetic base of the TBF trait of tea plants.

## Introduction

With over two billion cups of tea consumed by people across 160 countries every day, the tea plant (*Camellia sinensis*) is an economically important crop that is cultivated globally [1]. In 2019, over 9.29 million tons of tea were harvested from 8.26 million hectares of tea plant fields distributed in 48 countries (FAOSTAT, https://www.fao.org/faostat). The continuous genetic improvement of tea plants is an important task in major tea-producing countries, such as China, India, Japan, and Kenya. For that purpose, high-quality reference genomes of several tea cultivars have been assembled [2–4]. The biosynthetic pathways and main regulatory factors of important tea secondary metabolites, such as caffeine, theanine, and catechins, have also been studied intensively [5, 6]. However, in comparison with omics and molecular studies, progress in tea plant breeding methods and forward genetics has been relatively slow. The reasons for this lag include the following: (i) the long generation cycle and self-incompatibility of tea plants [7]; (ii) the fact that hand-pollination (HP) is time-consuming and labor-intensive; and (iii) the lack of a stable genetic transformation system [6]. Consequently, it is difficult to obtain large crossing populations for breeding selection and genetic analyses. Only a small portion of registered tea cultivars were bred by HP [8]. Currently, the methods of breeding tea plants still largely rely on individual selection from wild resources or sexual populations [9].

In addition to the specialized secondary metabolites, the timing of bud flush (TBF) in the spring is also among the most critical agronomic traits in tea plant breeding. Tea cultivars with an early TBF are highly valued for cultivation. Tea plants not only avoid numerous diseases and pests during the relatively low temperatures of spring but also accumulate a higher quantity of metabolites that are critical for their quality [10]. Early TBF tea cultivars can be sold earlier in the season, and they have a longer spring harvest time, resulting in a higher price and yield of spring tea.

The TBF of tea cultivars in the same environment can vary by up to 40 days [11]. However, the genetic control of this critical trait remains largely unclear. Several studies investigated this question by comparing the levels of gene expression in tea buds or leaves in dormant and flushing states [11–13]. The use of suppression subtractive hybridization enabled Wang *et al*. [11] to compare the levels of gene expression in winter dormant and sprouting axillary buds in two tea cultivars with distinct TBF and identified 1287 unigenes that are involved in water metabolism and hormone regulation among others. More recently, high-throughput transcriptome sequencing of tea buds at the dormant and flush stages often reveal >10 000 differentially expressed genes (DEGs) [12, 13]. However, this large number of DEGs has made it difficult to discern the causal genes that lead to the variation in TBF in tea plants. A few studies have examined the TBF trait of tea plants using quantitative trait locus (QTL) mapping and genome-wide association studies (GWAS) [10, 14]. To date, only two QTLs and one single-nucleotide polymorphism (SNP) that are significantly associated with TBF of tea plants have been reported.

**Figure 1:**
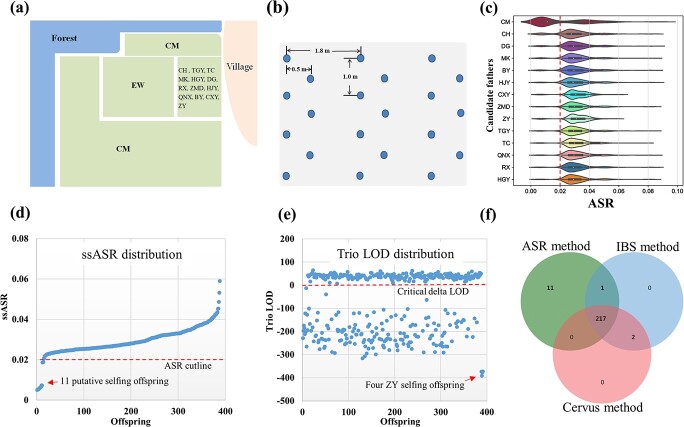
Distribution diagrams of tea plants used in this study and results of paternity assignments. **a** EW plantation for seed collection and the tea cultivars surrounding it. **b** EW offspring planting in the Mingshan experimental field. **c** ASR distribution for 14 candidate fathers. **d** Distribution of ssASR of each offspring with the 14 hypothetical parental couples. **e** Distribution of trio LOD scores of the 392 offspring when set with EW × CM as a parental couple. **f** Venn diagram showing the common results provided by the three paternity assignment methods for the EW × CM couple.

‘Emei Wenchun’ (EW) is a tea cultivar with an extra-early TBF, which was released in 2014 by Sichuan Province, China, and it is now widely cultivated in this province. The TBF of EW is 18–30 days earlier than that of ‘Fuding Dabaicha’ (FD), a commonly used control for tea breeding, and the ostensible endodormancy stage is almost absent in EW [13]. However, the genetic base and inheritance of the early TBF trait is largely unknown in EW. In addition, the yield, contents of free amino acids, and aroma traits of EW can be improved to obtain a better tea cultivar. Therefore, we designed an open-pollination (OP) breeding project between EW and other clonal cultivars with elite traits. Seeds collected from EW were sown, screened, and then recorded for their traits. A panel of offspring (*n* = 388) was selected for genetic analyses. The aims of this study were as follows: (i) to reconstruct the paternity of the 388 offspring based on large-scale SNP genotyping; (ii) to analyze the inheritance of the early TBF trait of EW by its offspring; (iii) to map the QTLs related to the TBF trait of tea plants; and (iv) to analyze the candidate genes that underlie the QTLs.

## Results

### The open-pollination breeding design

To integrate the early TBF trait of EW with the elite traits from other tea cultivars, EW was planted next to an existing test garden of tea cultivars in 2012 in Muchuan County (28°59′N, 103°53′E; Fig. 1a). In this test garden, there were high-fragrance oolong cultivars, including ‘Tiguanyin’ (TGY), ‘Mingke 1’ (MK), ‘Dangui’ (DG), ‘Ruixiang’ (RX), ‘Zimudan’ (ZMD), ‘Huangguanyin’ (HGY), and ‘Taicha 12’ (TC); high-amino acid cultivars, including ‘Baiye 1’ (BY, also called ‘Anji Baicha’), ‘Huangjinya’ (HJY), and ‘Qiannianxue’ (QNX); the high-anthocyanin cultivar ‘Ziyan’ (ZY); the yellowing cultivar ‘Chuanhuang 1’ (CH); and the small-leaf cultivar ‘Chuanxiaoye’ (CXY). In addition, the test garden was largely surrounded by the cultivar ‘Chuanmu 217’ (CM), which is a high-yield tea cultivar and widely cultivated in Muchuan.

More than 1500 open-pollinated seeds were harvested from EW and sown under greenhouse conditions in the autumn of 2017. Approximately 650 vigorous seedlings were transplanted to an experimental field in Mingshan County (30°12′N, 103°12′E); the distances between individuals are shown in Fig. 1b. Several agronomic traits were routinely observed, including the sprouting index (SPI), a trait closely related to TBF that is reported in this study.

### Genotyping results

A total of 407 samples, including EW, 14 candidate fathers, 388 EW offspring, and 4 putative ZY selfing offspring [15] (as controls) were genotyped using the specific-locus amplified fragment (SLAF) sequencing strategy. A total of 579.69 Gb of sequences of raw data were generated, and they contained 4.78 billion clean paired-end reads. An average of 95.27% reads had a Q30 quality score, indicative of good sequencing quality. Sequences of 4–103 bp of each clean read were extracted and mapped to the chromosome-level reference genome of ‘Shuchazao’ [3]. A total of 2 629 116 SLAF tags were developed, and 1 454 567 (55.3%) of them were polymorphic SLAFs. The average SLAF sequence depth of EW, candidate fathers, and all the offspring was 58.03, 30.74 and 11.57, respectively (Supplementary Data Table S1). A total of 12 336 977 SNPs were detected and the number varied from 1.63 to 5.95 million for each tested sample. These polymorphic SLAF tags and SNPs basically evenly covered all the 15 chromosomes of *C. sinensis* (Supplementary Data Figs S1 and S2). The heterozygous ratio of each sample varied from 2.53% to 9.52% with an average of 4.90% (Supplementary Data Table S1). The sequencing results also enabled the identification of 591 271 InDels compared with the reference genome (Supplementary Data Fig. S2). A total of 3047 of these InDels were found in the coding sequence regions. The SNP and InDel data were deposited in the Genome Variation Map in the National Genomics Data Center under accession number GVM000297.

### Paternity and population structure analysis

Three methods were utilized for paternity analysis to obtain common results with high confidence. A total of 229 218 SNPs that were called in ≥80% samples were used for the first two methods. First, abnormal SNP ratios (ASRs), defined as the percentage of loci where the allele of the offspring is absent in the two hypothetical parents, was used to exclude unlikely fathers. The ASR varied from 0.0042 to 0.0626 for the 388 EW offspring when EW was established as the mother and each of the 14 cultivars was considered to be a hypothetical father (Fig. 1c; Supplementary Data Table S2). In this study, we used ASR <0.02 as a cutline to exclude most false fathers. This cutline was determined based on the distribution of second smallest ASRs (ssASRs) of each offspring (Fig. 1d). Because each offspring has only one true father, and the ASR with the true parental couple should be lower than the ASR with false parental couples, the ASR cutline was set close to the lower limit of ssASRs. Based on our data, there are 14 ssASRs <0.02 and 11 ssASRs <0.008 (Fig. 1d). The rest of the ssASRs range from 0.02 to 0.06 and are primarily concentrated between 0.02 and 0.04. Interestingly, the 11 offspring that had an ssASR <0.008 also had an ASR <0.008 for all the 14 hypothetical parent pairs (Supplementary Data Table S2). They are probably the selfing offspring of EW since the ASR is always low because all the alleles of offspring could be found in the mother. Similarly, the four putative ZY selfing offspring controls also had a very low ASR when ZY was set as one of the parents. For the 14 candidate fathers, there were 11–229 offspring that met the ASR cutline. Among them, CM had the most offspring with ASR <0.02 (229), followed by CH (46), DG (25), MK (20), BY (19), and ZY (17) (Table 1).

**Table 1:** Number of offspring assigned to each parental couple by three methods.

**Putative parental couple**	**ASR method**	**IBS method**	**Cervus**	**Common assignments**
EW × CM	229	220	219	217
EW × CH	46	36	31	30
EW × DG	25	14	12	12
EW × MK	20	10	5	5
EW × BY	19	8	8	8
EW × HJY	15	4	4	4
EW × CXY	14	21	3	3
EW × ZMD	13	6	0	0
EW × ZY	17	10	2	2
EW × TGY	14	1	0	0
EW × TC	12	1	1	1
EW × QNX	12	3	1	1
EW × RX	11	0	0	0
EW × HGY	11	0	0	0
EW selfing	11^a^	11^b^	11	11

a Offspring that had ASR <0.008 for all 14 candidate fathers were regarded as EW selfing.

b Offspring with IBS >0.84 with EW were regarded as EW selfing.

Secondly, we calculated the identity by state (IBS) between offspring and candidate parents (Supplementary Data Table S3). IBS, also called the proportion of allele sharing, is often used as an important indicator in parentage analyses [16, 17]. For each offspring, the two candidate parents, including the mother EW, with the largest and second largest IBSs were selected as the possible parental couple. We used IBS <0.72 as a criterion to exclude unlikely father–offspring relationships. This criterion was selected because the IBS between the known mother–offspring relationships (388 offspring and their mother EW) varied from 0.7281 to 0.9402 (an average of 0.7624). After the filtering, 220 offspring were assigned to EW × CM. This number varied from 0 to 36 for other couples (Table 1). The 11 largest IBSs (varying from 0.8445 to 0.9402) were all scored by the 11 putative EW selfing offspring described above, but their IBSs with all the candidate fathers were <0.68. These results also imply that the 11 offspring originated from EW selfing. Similarly, the four putative ZY selfing offspring had a high level of IBS with ZY (>0.94).

Thirdly, the likelihood method, which estimates the possibility of paternity based on allele frequency and simulation analysis, was performed using Cervus 3.0 [18]. A set of high-quality and less linked SNPs (*n* = 572) were selected for this analysis. The critical delta logarithm of odds (LOD) with 99% confidence for paternity assignment was estimated to be 0 when the mother was known. There were 219 offspring that reached the critical delta LOD for the EW × CM couple (Fig. 1e; Supplementary Data Table S4). For other crosses, the number of offspring varied from 0 to 31. The 11 offspring that had ASR <0.008 for all candidate parent pairs were also assigned as EW × EW in the likelihood method when EW was added to the candidate father list.

The results of paternity assignment provided by these three methods were largely consistent. A Venn map of three methods for the EW × CM couple is shown in Fig. 1f. A total of 294 offspring out of the original 388 had common paternity assignments by the three methods (Table 1; Supplementary Data Table S5), and these putative parentage relationships were used for subsequent analysis.

**Figure 2:**
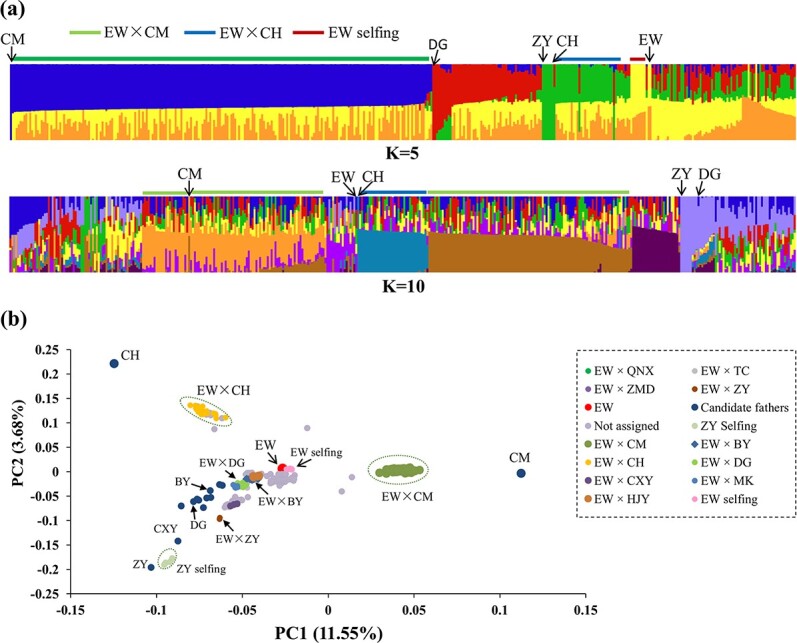
Results of population structure analyses of the samples tested in this study. **a** Population structure obtained from admixture when *K* = 5 and 10. **b** Scatter plot based on the first two principal components (PCs) of the principal component analysis.

The results of population structural analyses were consistent with the paternity assignments. When *K* = 5 was used in the admixture analysis, the colors were yellow and orange for EW, blue for CM, and green for CH (Fig. 2a). We could clearly distinguish the groups of EW × CM (bars that consisted of blue, yellow, and orange), EW × CH (green, yellow, and orange), and EW selfing (yellow and orange). When *K* = 10, the colors were more complicated, but it was still possible to discern the clusters of EW × CM and EW × CH from the whole populations. In the principal component analysis (PCA), the EW × CM offspring clustered in the middle of EW and CM, while the 11 selfing offspring of EW were adjacent to EW (Fig. 2b).

### Linkage map construction and evaluation

**Figure 3:**
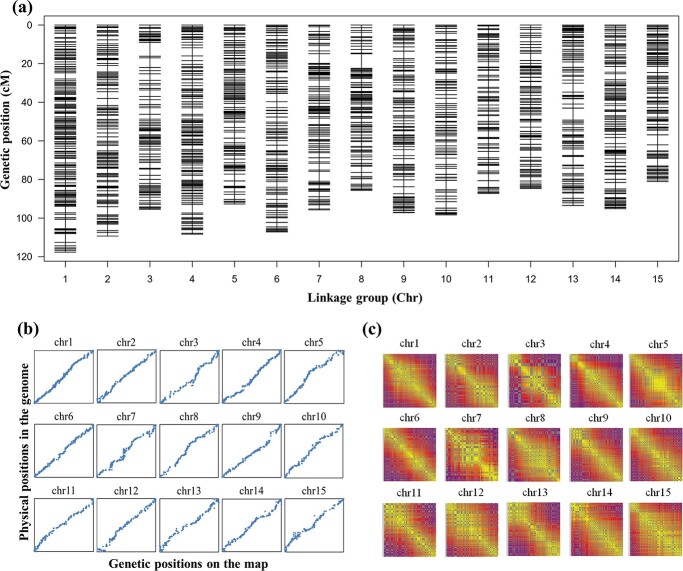
High-density linkage map constructed using genotypic data of the 217 full-sib offspring of EW × CM. **a** Distribution of SNP markers on the 15 linkage groups. **b** Collinearity analysis of the genetic positions of markers on the map and physical positions in the reference genome. **c** Heat maps showing distances and recombination rates between markers. The rates of recombination increase from yellow to purple. Gray indicates no available value for the recombination rate between the two markers.

The 217 full-sib offspring of EW × CM and their genotypes were used to construct a genetic linkage map. A total of 4244 SNP markers, including 53 distorted segregated markers (*P* < .01), were screened from the SNP data and successfully mapped into 15 linkage groups (Supplementary Data Table S6). The average genotype completeness of the mapped markers for each individual was 99.83%, and the average sequencing depths of the markers on the map for EW, CM, and the offspring were 226, 172, and 39.45, respectively, ensuring the genotyping accuracy of the mapped SNP markers. As shown in Fig. 3a and Table 2, the total genetic distance of the integrated map was 1449.19 cM, and the average distance between markers was 0.34 cM. The 15 linkage groups were numbered based on the 15 pseudo-chromosomes of the reference genome [3]. To compare the QTLs mapped in different populations, we also established the correspondence between linkage groups (Chr1–Chr15) in this study and those from previous studies [14, 19] (LG01–LG15) by BLASTing. The results are shown in Table 2 and Supplementary Data Table S7.

To evaluate the quality of the newly constructed map, we performed a collinearity analysis of the genetic positions of SNP markers on the map and physical positions in the reference genome. As shown in Fig. 3b, this map effectively covered the whole genome, and the marker orders of the linkage map were highly collinear with their physical positions. This was also supported by the Spearman coefficients in each group, which varied from 0.990 to 0.999 (Table 2). Figure 3c shows that the calculated rate of recombination of the two markers gradually increased as their distance increased, also indicating that the marker order on the map is correct overall.

### Phenotypic variation of the sprouting index trait among offspring

**Figure 4:**
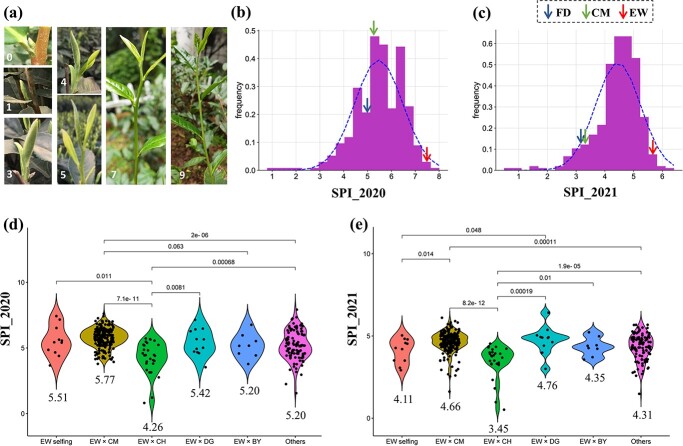
Phenotypic variation of SPI among the offspring in two consecutive years. **a** Representative pictures of tea buds with different SPIs. The SPI value is given in each picture. **b** and **c** Distribution of the 388 EW offspring based on SPI_2020 and SPI_2021. **d** and **e** Comparisons of average SPI values in different crosses. The significances of difference between two groups are indicated by *P* values from Kruskal–Wallis tests.

The TBF of tea plants is difficult to quantify and was substantially affected by the year’s weather. In this study, we observed the SPI of 5–10 representative overwintering buds and calculated the average SPI to represent the TBF of each offspring. A higher SPI indicates that the TBF was earlier (Fig. 4a). The SPIs were observed on 11 March 2020 (designated SPI_2020) and 20 February 2021 (SPI_2021). SPI_2020 varied from 0.8 to 8.0 (mean = 5.465), and SPI_2021 varied from 0.5 to 6.4 (mean = 4.457). The coefficients of variation were 18.5 and 17.6%, respectively. The results indicate that SPI had a wide segregation in the offspring population of EW. The difference between the earliest and the latest TBF among the offspring was estimated to be >30 days considering the speed of growth in the spring (5–6 days per leaf) [20].

The SPIs of EW, CM, and FD are indicated by arrows in Fig. 4b and c. Most of the offspring of EW had a lower SPI than EW. However, four individuals in 2020 and 10 in 2021 had an equal or higher SPI than that of EW. When compared with FD, 69.8% in 2020 and 92.3% in 2021 of the offspring had a higher SPI. Considering that FD is a tea cultivar with early TBF [8], it can be presumed that most of the offspring had an early or extra-early TBF.

It is worth comparing the SPI among different crosses to deduce the impacts of male parents. As shown in Fig. 4d and e, the offspring of EW × CH recorded the lowest average SPI in both years, which was significantly lower than those of EW × CM and EW × DG. This is reasonable since CH is a late TBF cultivar based on the breeding records. Somewhat unexpectedly, the average SPI of the 11 EW selfing offspring was apparently lower than that of EW and even significantly lower than those of EW × CM and EW × DG in 2021.

### QTL mapping results

**Figure 5:**
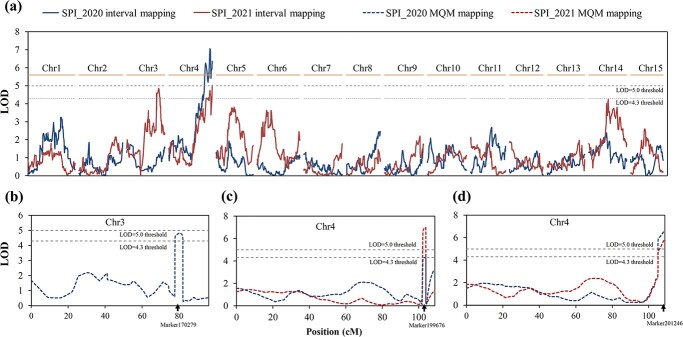
QTL mapping results with the SPI trait in the EW × CM full-sib population. **a** LOD value for 2020 and 2021 covering the whole genome by interval mapping. **b**–**d** MQM mapping results in Chr3 and Chr4. Genome-wide LOD thresholds at 99 and 95% for QTL claiming are shown by dotted lines. Cofactors used for MQM mapping are indicated by arrows in **b**–**d**.

Interval mapping in MapQTL6.0 software [21] was performed to map QTLs with the SPI data. Based on permutation tests, the genome-wide LOD thresholds at 99 and 95% confidence for QTL claiming were 4.3 and 5.0, respectively. As shown in Fig. 5a, there was one region on Chr4 (89.0–108.3 cM) that was higher than the 99% LOD threshold in the SPI_2020 data. An LOD peak at 7.06 had a phenotypic variation explained (PVE) of 14.2% at 102.821 cM. This region was also significant with SPI_2021. However, the highest LOD was scored at 108.362 cM with a PVE of 10.6%. A significant position at 95% on Chr3 was also detected in SPI_2021 (LOD peak = 4.86 and PVE = 10.2%); but this position was not significant in SPI_2020.

**Table 2:** Details of the linkage map constructed in this study.

**Linkage group**	**Marker number**	**Total distance (cM)**	**Average distance (cM)**	**Percentage of gaps ≤5 cM**	**Distorted marker**	**Spearman rank coefficient** ^**a**^	**Previous linkage group** ^**b**^
Chr1	492	117.63	0.24	100.00	0	0.998	LG03
Chr2	357	109.32	0.31	100.00	3	0.997	LG08
Chr3	230	95.46	0.42	99.13	2	0.994	LG06
Chr4	349	108.36	0.31	100.00	6	0.999	LG01
Chr5	274	92.70	0.34	100.00	12	0.997	LG04
Chr6	355	107.15	0.30	100.00	0	0.998	LG02
Chr7	278	95.75	0.35	99.64	11	0.992	LG09
Chr8	241	85.69	0.36	99.58	0	0.998	LG05
Chr9	253	97.22	0.39	100.00	0	0.999	LG10
Chr10	225	98.34	0.44	100.00	0	0.999	LG12
Chr11	223	87.25	0.39	100.00	3	0.996	LG11
Chr12	261	84.67	0.33	100.00	0	0.995	LG14
Chr13	229	93.49	0.41	99.56	0	0.990	LG07
Chr14	244	95.12	0.39	100.00	16	0.998	LG13
Chr15	233	81.04	0.35	99.57	0	0.993	LG15
Total	4244	1449.19	0.34	99.83	53	0.998	—

a Spearman rank correlation coefficient of the genetic positions of SNP markers on the map and physical positions in the reference genome.

b Linkage groups reported in Tan *et al*. [14] and Xu *et al*. [19].

To detect whether there are two or more neighboring QTLs on Chr3 and Chr4, we performed a multiple-QTL model (MQM) analysis using Marker170279 on Chr3 and Marker199676 or Marker201246 on Chr4 as cofactors. As shown in Fig. 5b–d, the supportive interval narrowed to the cofactors in MQM analysis, but no position elsewhere was found to exceed the LOD thresholds. Thus, we considered only one QTL to be related to the SPI on each of the two linkage groups and designated them *qSPI3* and *qSPI4*, respectively (Table 3).

**Table 3:** Details of the two major QTLs detected by interval mapping.

**QTL**	**Trait data**	**Peak LOD**	**Position (cM)**	**PVE**	**Nearest marker**	**COI (cM)**	**Flanking markers**
*qSPI3*	SPI_2021	4.86	79.972	10.2%	Marker170368	74.618–84.198	Marker170391 **—** Marker171932
*qSPI4*	SPI_2020	7.06	102.821	14.2%	Marker199676	89.078–108.362^a^	Marker197226 — Chr end
	SPI_2021	5.06	108.362	10.6%	Marker201246		

a For *qSPI4*, the highest LOD was recorded at neighboring but different positions in the two years. We selected a wide COI to cover both positions.

Kruskal–Wallis analyses in MapQTL6.0 were performed to detect the markers that significantly related to the SPI. In addition to the markers in the regions on *qSPI3* and *qSPI4*, four SNP markers on the other groups met the detection criteria and were reported as minor QTLs (designated *mqSPI*; Table 4). Among them, Marker212117 (Chr5) and Marker127595 (Chr14) were significant at *P* < .005 in both years, while Marker313352 (Chr9) and Marker144884 (Chr15) were only significant at *P* < .001 in 2021. In the interval mapping, the LOD at Marker212117 (3.48) and Marker127595 (3.77) in 2021 also met the linkage group LOD thresholds at 95% confidence (3.2). The *K*-values, genotypes of parents and offspring, breeding-favorable genotypes (higher average SPI), and the average SPI of different genotypes for each minor QTL are shown in Table 4.

**Table 4:** SNP markers significantly related to SPI detected by Kruskal–Wallis analyses.

**QTL**	**Marker ID**	**Position (cM)**	**Trait data**	K*	**Significance**	**EW × CM genotypes**	**Offspring genotype**	**Average SPI of corresponding genotypes in EW × CM offspring** (*n* = 217)	**Average SPI of corresponding genotypes among other offspring** (*n* = 171)
*qSPI3*	Marker170368	79.972	SPI_2020	3.500	^*^	GT × GG	GG:GT	5.67: 5.88	5.03: 5.05
			SPI_2021	13.871	^******^			4.49: 4.85	4.17: 4.21
*qSPI4*	Marker199940	105.092	SPI_2020	22.412	^*******^	AG × AG	AA:AG:GG	5.39: 5.76: 6.01	4.70: 5.04: 5.44
			SPI_2021	18.770	^*******^			4.38: 4.62: 4.87	4.19: 3.97: 4.58
*mqSPI5*	Marker212117	35.436	SPI_2020	8.586	^****^	CT × TT	CT:TT	5.90: 5.62	5.10: 5.05
			SPI_2021	9.805	^****^			4.81: 4.50	4.22: 4.08
*mqSPI9*	Marker313352	96.926	SPI_2020	2.40	ns	CT × CC	CT:CC	5.84: 5.68	5.21: 4.91
			SPI_2021	11.804	^*****^			4.75: 4.55	4.23: 4.14
*mqSPI14*	Marker127595	44.789	SPI_2020	8.001	^****^	AA × AT	AA:AT	5.61: 5.91	4.92: 5.44
			SPI_2021	8.243	^****^			4.53: 4.78	4.06: 4.43
*mqSPI15*	Marker144884	39.697	SPI_2020	0.348	ns	AG × AG	AA:AG:GG	5.77: 5.78: 5.72	5.18: 4.90: 5.10
			SPI_2021	16.320	^******^			4.76: 4.71: 4.40	4.14: 4.09: 4.38

Significance levels: ^*^ .1, ^***^ .01, ^****^ .005, ^*****^ .001, ^******^ .0005, ^*******^ .0001; ns, not significant.

Breeding-favorable alleles in the parents and genotypes in offspring with higher SPI are underlined as highlights.

We analyzed these genotype–phenotype relationships in the remaining 171 half-sib offspring except for the 217 offspring of EW × CM. Consistent results were observed for the other five markers as shown in Table 4, except for Marker144884, which was discarded from further analyses. The breeding-favorable genotypes in the EW × CM full-sib population also scored higher SPI in the half-sib offspring in both years (Table 4). For example, there are three genotypes (AA, AG, and GG) for Marker199940 in the offspring, and the highest average SPI was always recorded for the GG types in both EW × CM full-sib and half-sib offspring. The results indicate that the relationships between the SPI phenotype and genotypes at the five markers are relatively stable.

### Candidate gene analyses of the QTLs

For *qSPI3* and *qSPI4*, the peak LOD-1 regions were used as the confidence intervals (COI) for candidate gene searc hing. The COI for *qSPI3* was 74.618–84.198 cM, which corresponded to a 13.48-Mb sequence in the genome of ‘Shuchazao’ (Table 5). The COI for *qSPI4* was more complicated since the LOD peaked at different positions during the two years. We selected 89.078–108.362 cM of Chr4 as the COI for *qSPI4* to cover the LOD peaks in both years. The COIs of the minor QTLs detected by the Kruskal–Wallis analysis were determined by the marker’s physical position ±5 Mb. As shown in Table 5 and Supplementary Data Table S8, 1960 genes were predicted in the five COIs.

**Table 5:** Predication of candidate genes underlining the QTL or markers that related to SPI.

**QTL**	**Genome position of COI**	**Predicted gene number (newly annotated gene number)**	**Differentially expressed gene number**	**GO/KEGG/NR annotation**	**Key candidate genes** ^**a**^
*qSPI3*	142.305–155.782 (13.48 Mb)	241 (57)	34	25/24/32	CSS0016341 (*SLEEPER*); CSS0018684 (*BRI1*); CSS0029008 (*WRKY*)
*qSPI4*	157.841–196.299 (38.45 Mb)	1159 (216)	230	179/143/212	CSS0000275 (*PILS6*); CSS0001166 (*ERF*); CSS0006097 (*PP2C*); CSS0009754 (*WRKY*); CSS0010958 (*DELLA*); CSS0013463 (*PILS6*); CSS0015228 (*PYL*); CSS0027597 (*PYL*); CSS0042437 (*TIP*); CSS0044037 (*PIP*); NG12184 (*SLEEPER*); NG12339 (*DOF*)
*mqSPI5*	92.586–102.586 (10 Mb)	203 (67)	38	25/20/29	CSS0041392 (*PIP*)
*mqSPI9*	158.022–165.547 (7.53 Mb)	123 (29)	24	12/14/22	CSS0043965 (*BTB/POZ*); CSS0036456 (DNA meth); CSS0027936 (*F-box*); CSS0019824 (DNA meth)
*mqSPI14*	59.490–69.490 (10 Mb)	234 (45)	44	31/25/41	CSS0001638 (*BRI1*); CSS0013568 (*ERF*)
Total	85.98 Mb	1960 (414)	370	272/226/336	22

See text for abbreviations of candidate gene names and categories.

a Gene names that start with ‘NG’ are newly predicted genes from the RNA-Seq data [13].

To further narrow the range of candidate genes, we analyzed and compared the levels of expression of the genes located in the COIs. The RNA-Seq data [13] that were previously reported from the dormant and flushing buds of EW and ‘Chuancha 2’ (CC, a control with relatively late TBF) were reanalyzed based on the reference genome. A total of 370 of the 1960 genes in the COIs were found to be significantly differentially expressed in the comparisons of EW-D versus CC-D and EW-D versus EW-F (-D indicates dormant buds, while -F indicates flushing buds) (Supplementary Data Table S9). These DEGs are more likely to be the causal genes of QTLs, and therefore we focused on them in more detail. Among the 370 DEGs, 272, 226, and 336 had annotations in Gene Ontology (GO), Kyoto Encyclopedia of Genes and Genomes (KEGG) and non-redundant protein sequence database (NR), respectively. The annotations were used to search the key candidate genes based on references that indicate particular genes that are related to plant dormancy/germination regulation. Finally, 22 genes were preliminarily selected as key candidate genes for the five QTLs (Table 5). These genes included transcript factors, such as *WRKY* and *Ethylene-Responsive transcription factor* (*ERF*); genes related to plant hormones, such as auxin efflux carrier family protein *PILS6*, abscisic acid (ABA) signaling components *Type 2 C Protein Phosphatases* (*PP2C*), *Pyrabactin Resistance-Like* (*PYL*), and *DELLA*, *Brassinosteroid Insensitive 1* (*BR1*); aquaporin water channel genes *PIP* and *TIP*; *zinc finger BED domain-containing protein RICESLEEPER 2-like*; and genes related to F-box proteins and DNA methylation. Their levels of expression in the bud samples of EW-D, EW-F, CC-D, and CC-F are shown in Supplementary Data Fig. S3.

## Discussion

### Paternity reconstruction based on genotypes can assist breeding and genetic analysis in tea plants

Clear pedigree information is important in breeding, genetic studies, germplasm conservation, and understanding the histories of cultivars [22]. However, the majority of tea cultivars have no pedigree information since they were bred by individual selection from sexual populations or OP crosses [8]. HP in tea plants is notoriously time- and labor-consuming for two reasons. First, tea plants need ~450 days from the differentiation of flower buds before the seeds mature [7]. Secondly, 65–99% of the young fruits will drop naturally before maturity, which primarily occurs during the winter, and each fruit that matures has only one to three seeds [23]. Therefore, to obtain a full-sib population with one or two hundred individuals, tea breeders often need to pollinate thousands of flowers.

In this study, we designed an OP breeding project using EW as the female parent. A total of 388 offspring with breeding potential were selected for genotyping, and the genotypic results were successfully used to reconstruct paternity for 75.8% of the offspring. These results indicate that as an alternative to the time- and labor-intensive HP, a designed OP breeding project combined with paternity reconstruction is an effective strategy to obtain new tea hybrids of known parents. This strategy is now attractive for tea breeding since genotyping has become less expensive.

A large full-sib population is not only important for breeding selection; it is also critical for genetic mapping in tea plants. Such populations were usually developed from massive HP, and only a few full-sib populations with >150 individuals have been reported in tea plants [19, 24–26]. In this study, as expected, paternity reconstruction found that more than half of the OP offspring (55.9%) were from one crossed couple (EW × CM). This putative full-sib population (*n* = 217) was used directly to construct a high-density linkage map. To our knowledge, this map is one of the most high-quality linkage maps in *C. sinensis* regarding population size and the number of markers. It can be used for the QTL mapping of TBF, as well as other agronomic traits of tea plants. Furthermore, as an advantage of a perennial crop, more seeds can be collected from the maternal EW plants each year at low cost. For example, the population size of EW × CM can be further expanded to >1000 when needed for QTL fine-mapping. To increase the ratio of EW × CM (or other crosses) among the OP seeds, we can keep the flower buds of the two parents, while reducing those of the other cultivars by heavy pruning.

### Genetic architecture of the TBF trait in tea plants

TBF is an economically important trait in tea plants. This trait is related to the regulation of winter dormancy, which is an aspect of perennial plants that is the focus of a substantial amount of research. Winter dormancy is a complex trait that is regulated by both external environmental factors, primarily sunlight, and temperature, and internal genetic factors, such as phytohormones, transcription factors, and functional genes [27]. QTL mapping is a common method used to study such a complex trait. We previously mapped two QTLs related to the TBF of tea plants (*qTBF1-1* and *qTBF1-2*) to LG01 in the full-sib population of ‘Longjing 43’ × ‘Baihaozao’ [14]. Interestingly, the two QTLs are in the same linkage group as *qSPI4* in this study (Chr4). The closest marker to *qTBF1-1* (CsFM1875, located at 179.99 Mb of Chromosome 4) was within the COI of *qSPI4*. The closest marker of *qTBF1-2* (CsFM1390, located at 133.37 Mb of Chromosome 4) was also close to *qSPI4*. These results indicate that, among the many genetic loci that may control the TBF of tea plants, *qSPI4* or *qTBF1-1* may be a major one and is prevalent in different tea accessions.

In addition to *qSPI4*, this study also mapped *qSPI3* as a major QTL and three minor QTLs. Although not as significant as *qSPI4*, their effects appeared to be stable over 2 years in EW × CM full-sib and the EW’s half-sib populations (Table 4). Wang *et al*. [10] also reported SNPs associated with the TBF of tea plants, and the most significant SNP (Sc0002405-269473) was characterized in detail [10]. This SNP was localized to Chromosome 13 of the reference genome and did not overlap with any of the QTLs identified in this study. Taking these results together, one can conclude that there are many genetic loci with a small effect that control the TBF of tea plants. These loci are distributed on Chr4, Chr13, Chr3, Chr5, Chr9, and Chr14 among others.

A wide segregation of SPI was observed among the offspring of EW, which is probably determined by the highly heterozygous genome [2] and the complex genetic architecture of the trait as described above. Nevertheless, a few of the offspring (1.0–2.6%) had a similar or an even larger SPI than EW, indicating that it is a promising method to obtain new breeding materials with extra-early TBF from the offspring of EW. The QTLs and their linked markers reported from this study will be useful for the early selection of individuals with early TBF.

### Genomic and transcriptomic data contribute to QTL candidate gene analyses

The publication of chromosome-level reference genomes of tea plants [2–4] greatly facilitated genetic studies on this crop, including the QTL candidate gene analyses described in this study. The genome fragment was framed using the positions of flanking markers of QTL, and a search of candidate genes within its COI became possible. In this study, the physical distances of COIs varied from 7.53 to 38.45 Mb. They are still too long for gene identification since they harbor 123–1159 predicted genes. Owing to the limited mapping population size and the large genome size (>3 Gb) [3], it is difficult to fine-map a QTL, such as within 1 Mb of the sequence or less, in tea plants. Therefore, we used the transcriptome data of EW and CC [13] to help search for candidate genes. We assumed that the candidate genes would be differentially expressed between the dormant and flushing buds of EW (EW-D versus EW-F) or between the dormant buds of two cultivars with different TBF (EW-D versus CC-D). Considering only the DEGs, the candidate gene number within each COI was narrowed to dozens. Analyses of gene annotations and the literature further point to several key candidate genes (Table 5). Although this filtering process cannot ensure the accurate identification of the real causal genes, it provides clues for the next step of verifying gene function. In-depth analysis of the variation and expression of these genes in two parents and offspring with different SPI traits is recommended in future studies.

Among the final key candidate genes in Table 5, there are two *WRKY* and two *ERF* transcription factors. *WRKY* transcription factors are well known components in ABA signaling. Therefore, they are likely to participate in the regulation of plant dormancy and germination [28]. Both *WRKY*s identified here (CSS0029008 and CSS0009754) were expressed at significantly higher levels in EW-D than CC-D. A *Populus tremula ERF* (designated *Early-Bud Break 1*, *PtEBB1*) was proven to confer the early bud break phenotype [29]. In addition to transcription factors, there are many genes related to hormones in the key candidate gene list. For example, auxin efflux carrier family protein *PILS6* (CSS0000275, CSS0013463); ABA signaling components *PP2C* (CSS0006097), *PYL* (CSS0015228, CSS0027597), and *DELLA* (CSS0010958); and *BR1* (CSS0018684, CSS0001638). These hormone-related genes have been reported to regulate growth and dormancy in a wide range of plants [30, 31].

DNA methylation is considered to be an important factor in the regulation of plant dormancy [12, 32]. A KELCH F-BOX gene in the COI of Marker313352 (CSS0027936) was expressed at significantly higher levels in EW than CC. This gene was highly similar to *AtCFK1* in *Arabidopsis*, which participates in the regulation of DNA methyltransferase protein abundance [33]. Interestingly, there are two ‘increased DNA methylation 3-like isoform’ genes (CSS0036456 and CSS0019824) in the same COI region, and they were expressed at significantly higher levels in CC than in EW. Aquaporin water channels have also been reported to be related to the germination of seeds in rice [34] and winter dormant bud flushing in peach [35]. Two such genes (CSS0042437 and CSS0044037) were found in the COI of *qSPI4*, and they were expressed at significantly higher levels in EW-D than CC-D.

### Partial breakdown of self-incompatibility in tea plants

Self-incompatibility is common in flowering plants and considered to be an important system to prevent inbreeding. Tea plants are regarded as being selfing-incompatible with late-acting self-incompatibility [36]. However, self-incompatibility has been weakened or inactivated in a few tea clones. Wachira and Kamunya [37] reported pseudo-self-incompatibility in a genotype designated ‘TRFK K/purple’. We also found by paternity reconstruction that ZY is self-compatible with a high selfing rate (~29.5%) in the OP offspring [15]. In this study, we found that 11 offspring (2.5%) were from the selfing of EW by paternity reconstruction. This ratio is much lower than that of ZY, suggesting that the self-incompatibility is partially inactivated in EW. However, the selfing ratio could be underestimated owing to the selection of vigorous individuals for genotyping.

Breeders and researchers have long been challenged by the self-incompatibility and high degree of heterozygosity of tea plants [38, 39]. The 11 putative EW selfing offspring identified here have a heterozygous ratio that varied from 2.53 to 4.64% (average 3.15%), significantly lower than their mother (6.82%) and other crossing offspring (average 4.90%; Supplementary Data Table S1). Therefore, they may have applications in breeding and genomic studies [15]. The average SPI of the 11 selfing offspring was lower than that of EW in both years and even significantly lower than those of other crosses in 2021. This could partially be attributed to the strong selfing depression effect in tea plants [15].

### Conclusions

In this study, we report the genetic analysis of the early TBF trait of tea plants using EW and its offspring from an OP breeding project. Large-scale SNP genotyping of the offspring and candidate parents enabled the identification of putative paternity for most of the offspring. This lays a foundation for the construction of a high-density map, the identification of QTLs related to SPI, and the finding of partial breakdown of self-incompatibility in EW. Furthermore, 22 key candidate genes that underlie the QTLs were preliminarily screened using the reference genomic and transcriptomic data. The results will be useful for selecting tea individuals with early TBF, understanding the genetic control of this trait, and cloning the genes. The research strategy used in this study is also recommended to increase the speed of crossbreeding and genetic mapping in tea plants.

## Materials and methods

### Plant materials

The parental garden of EW and 14 other clonal tea cultivars shown in Fig. 1a was in Muchuan County in Sichuan Province, China. The offspring population used in this study were derived from EW seeds, which were directly collected from clonal EW plants in the autumn of 2017. At the time, EW was 5 years old, while the other 14 tea cultivars were 2–3 years older. Other clonal and sexual tea plantations also existed within 1 km but were relatively far from this site. Seeds were sown in a greenhouse (22 ± 2°C, 12 h/12 h light/dark) in November 2017. The vigorous seedlings were transplanted to the experimental tea field in Mingshan County, Sichuan Province, in February 2018. The experimental field was managed using the conventional management methods for juvenile tea plants.

Young leaf samples of 388 individuals were collected for genotyping. Leaf samples of EW and the 14 candidate fathers that closely surrounded EW were collected from the parental plants. In addition, four selfing individuals of ZY obtained in our previous study [15] were also genotyped and analyzed as external controls. All the leaf samples were quick-frozen in liquid nitrogen, transported in dry ice and stored at −80°C until the DNA was extracted.

### Genotypic analysis

The total genomic DNA of the samples was extracted using the CTAB method. After quantity and quality evaluation, the DNA of each sample was digested with HaeIII (TaKaRa, Dalian, China). SLAF libraries were constructed as described by Sun *et al*. [40]. SLAF-Seq was then performed on an Illumina NovaSeq 6000 (Illumina Inc., San Diego, CA, USA) at the Biomarker Technologies Corporation (Beijing, China). The raw reads obtained were screened for barcode adapters and reads with >10% N to obtain clean reads. The sequence of 4–103 bp of each clean read was extracted and mapped to the reference genome using the Burrows–Wheeler Aligner (BWA) [41]. GATK [42] and SAMtools were then used to identify SNPs and InDels, and the common results called by the two tools were used for further analysis.

### Paternity reconstruction and population structure analyses

A total of 229 218 SNPs detected in >80% samples were obtained after filtering the raw SNP calls with ‘—max-missing 0.80’ using VCFtools [43]. This set of SNPs, with a mean sequencing depth of 19.34, was used to calculate the ASR and IBS, as well as admixture and PCA analyses. The ASRs of 14 parent combinations (EW × one of the 14 candidate fathers) were calculated for each individual. An abnormal SNP was defined as the allele(s) of the offspring that was absent in EW and the hypothetical father. The IBS between the samples was calculated using PLINK 1.90 [44]. Admixture [45] was used to analyze the population structure based on the maximum-likelihood method with 10 000 iterations. A PCA was performed using EIGENSOFT software [46].

The SNPs were further filtered using VCFtools with the following criteria: minor allele frequency (—maf 0.3), Hardy–Weinberg equilibrium (—hwe 0.05), minimum sequencing depth (—minDP 5), data integrity (—max-missing-count 20), and tight linkage (—thin 3 000 000). The remaining SNPs (*n* = 572) were used to perform parentage assignment with Cervus3.0 [18]. To determine the critical delta trio LOD score with 99% levels of confidence, a simulation paternity analysis was performed with the following parameters: proportions of loci genotyped (.99), mistyped (.01), and parents sampled (.75); and the numbers of offspring (10 000), candidate fathers (15), and minimum typed loci (500). Paternity assignments were performed with the known mother (EW). A replicate sample of the EW (designated EW2) was added to the candidate father list to detect possible selfing.

### Linkage map construction

The genotype data of EW × CM full-sib population were prepared and filtered with the following standards: (i) SNPs with a sequencing depth <10× in the two parents; (ii) SNPs with a sequencing depth <2× in any one of the offspring; and (iii) SNP markers with significantly distorted segregation (χ^2^ test, *P* < .01). The remaining SNPs were used to calculate the modified LOD (MLOD) values and filter the SNP with MLOD <5 between any other markers. The SNPs were arranged from small to large according to the MLOD value. The markers with the highest MLOD value were in the same linkage group. The order of markers and genetic distance between them within each linkage group were arranged and calculated using HighMap [47]. To fill some large gaps in the map, some SNPs with distorted segregation or a sequencing depth <2× in a few offspring were also used to refine the linkage map. Spearman’s rank correlation coefficient was calculated using the Statistics::RankCorrelation Perl module (http://www.cpan.org/) to evaluate the collinearity between each linkage group and the physical map.

The correspondence between linkage groups of *C. sinensis* (Chr1–Chr15) in this study and those of previous studies (LG01–LG15) were established by BLASTing at TeaPGDB [48]. The unigene sequences that harbored the simple sequence repeat (SSR) markers on a previous linkage map with at least three markers on each group were used as query sequences, while the genome of ‘Shuchazao’ was set as the query database. The SNP (Sc0002405–269 473) reported in Wang *et al*. [10] was mapped to a chromosome using the same method.

### Phenotyping of the sprouting index trait

The SPI for each bud was recorded with a number, where 0 represents dormant, 1 represents start to sprouting (bud with a green tip), 2 represents scales open, 3 represents fish-leaf open, 4 represents one bud and one leaf, 5 represents one bud and two leaves, and 6 represents one bud and three leaves and so on (Fig. 4a). Since the apical buds and tender shoots were pruned in November of 2019 and 2021, the top two axillary buds of each robust branches were scored for SPI. The SPI of each individual was the mean of at least 5–10 overwintering buds. Simultaneously, the SPI values of EW, CM, and FD planted near the offspring field were also observed. The data were collected on 11 March 2020 and 20 February 2021. The date was chosen when the majority of offspring had reached ‘one bud and one leaf’ status. However, the time of observation in 2020 was relatively late because of the COVID-19 travel ban. The buds of >10 offspring had paused after flushing four or five leaves on 11 March 2020.

### QTL mapping

QTL mapping was performed using MapQTL6.0 [21]. Genome-wide LOD thresholds of 99 and 95% to declare the QTLs were calculated using a permutation test of 1000 replications. The PVEs of the QTLs detected were calculated and a QTL with >10% PVE was defined as the major QTL. To detect whether there are two or more neighboring QTLs on Chr3 and Chr4, MQM analysis was performed using cofactors suggested by automatic cofactor selection. Furthermore, a Kruskal–Wallis analysis was performed to detect minor QTLs related to the SPI. Markers that were significant at *P* < .001 in at least one year or *P* < .01 in both years in the Kruskal–Wallis analysis were reported. Only the marker with highest *K*-value was reported from each linkage group.

### Transcriptomic data reanalysis

The previously reported RNA-Seq data [13] from bud samples of EW and CC were mapped to the new genome of ‘Shuchazao’ using HISAT2 [49]. The mapped reads were assembled and compared with the original annotations of the genome by StringTie [50]. The transcript regions without annotation are defined as novel genes (Supplementary Data File 1). The number of fragments per kilobase of transcript per million mapped fragments (FPKM) was calculated to measure the level of gene expression by StringTie using a maximum flow algorithm. The analysis of differential expression was processed using DESeq2 [51]. The criteria for the DEGs were set as fold change ≥2 and false discovery rate <.01.

## Acknowledgements

This work was funded by National Natural Science Foundation of China (31800589), the Department of Science and Technology of Sichuan Province (2021YFYZ0025) and the National Innovative Experiment Plan for College Students (202010626132).

## Author contributions

L.T., D.C., L.W., Q.L., D.Z., X.H., Y.F., S.C., Y.Z., and W.C. performed the experiments and data analyses. W.W., X.Y., and Y.Y. contributed to plant management and phenotype data collection. L.T. wrote the paper. Q.T. and P.L. coordinated the project. All authors read and approved the final manuscript.

## Data availability

The SNP and InDel data were deposited in the Genome Variation Map in the National Genomics Data Center under accession number GVM000297. The details of paternity assignment, information on markers on the linkage map, annotations and expression levels of genes within the COIs of QTLs are provided in the Supplementary Data tables.

## Conflict of interest

The authors declare that they have no conflict of interest.

## Supplementary data

Supplementary data is available at *Horticulture Research* online.

## Supplementary Material

uhac086_suppl_data
