# Supplementary figures and images for: Genetic analysis of the early bud flush trait of tea plants (Camellia sinensis) in the cultivar ‘Emei Wenchun’ and its open-pollinated offspring
Source: Hortic Res. 2022 Apr 21;9:uhac086. doi: 10.1093/hr/uhac086 (PMC9178331; doi:10.1093/hr/uhac086)

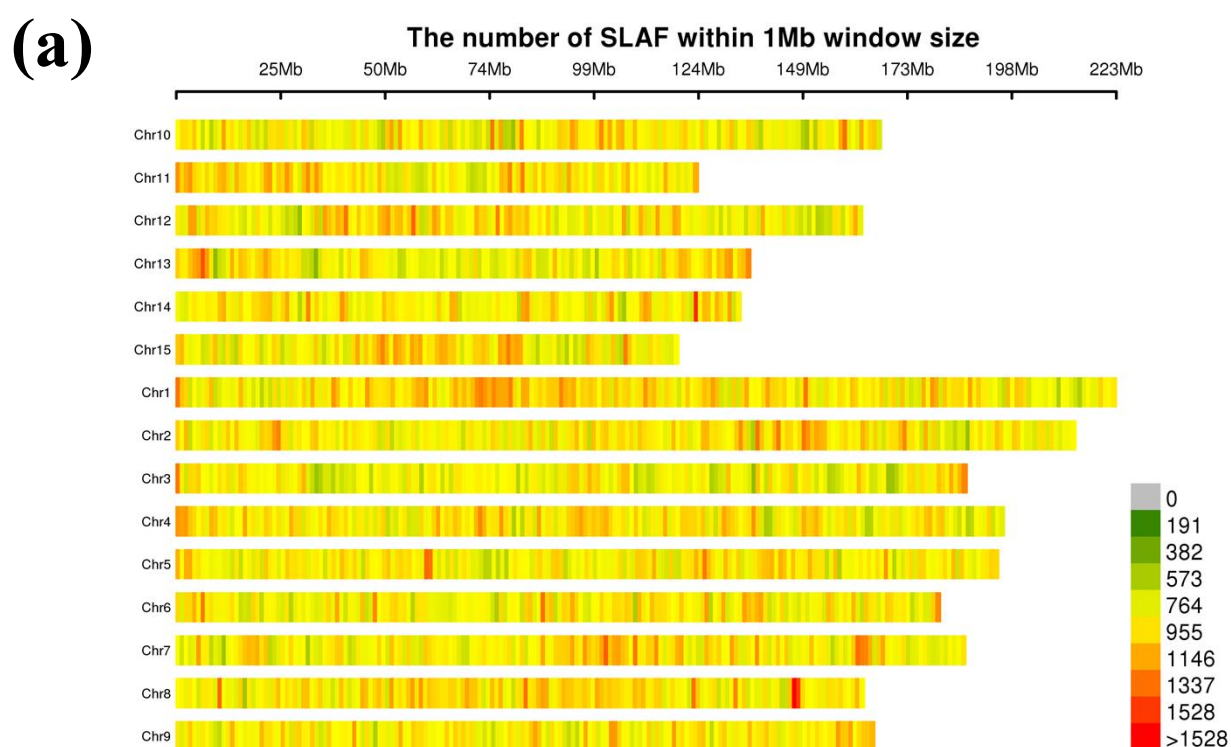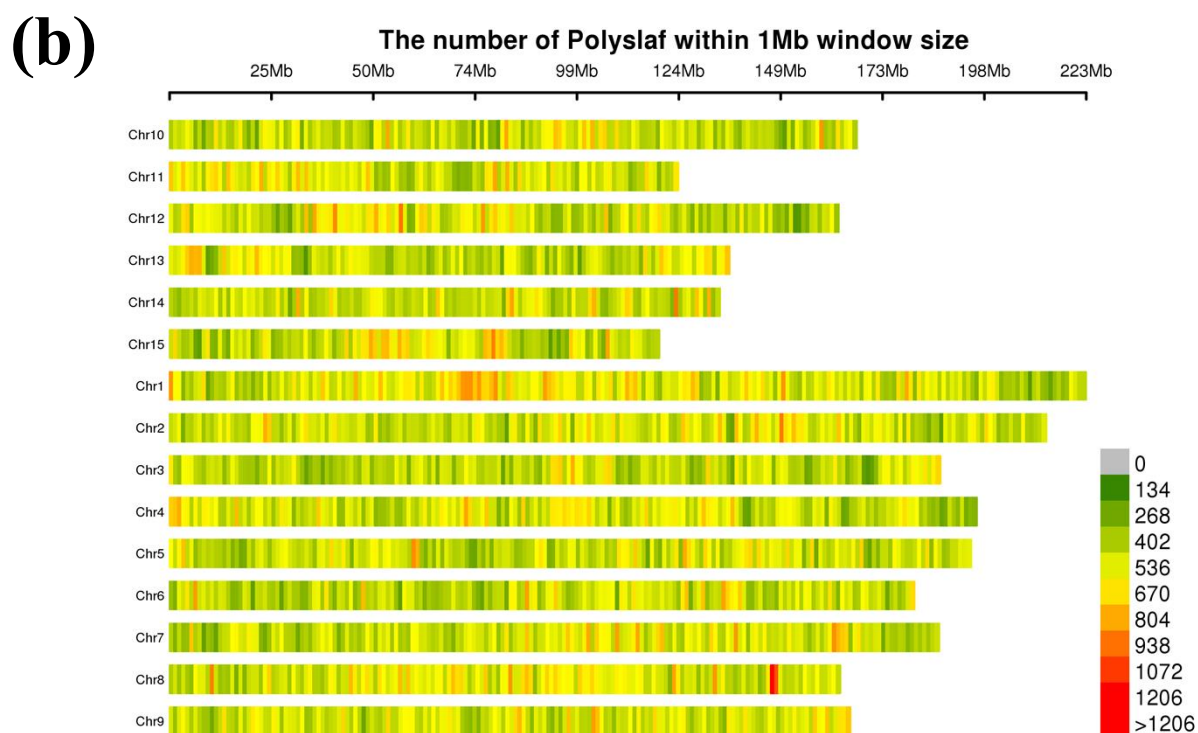

(a)

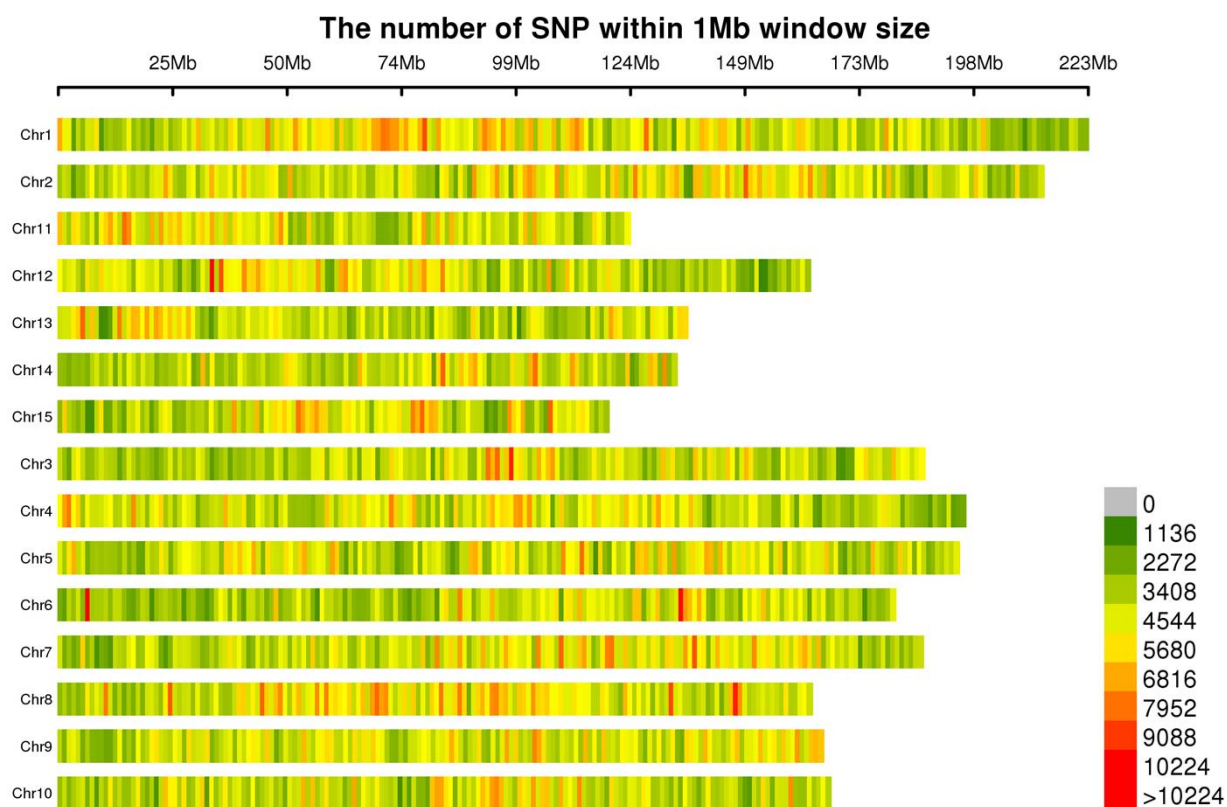

(

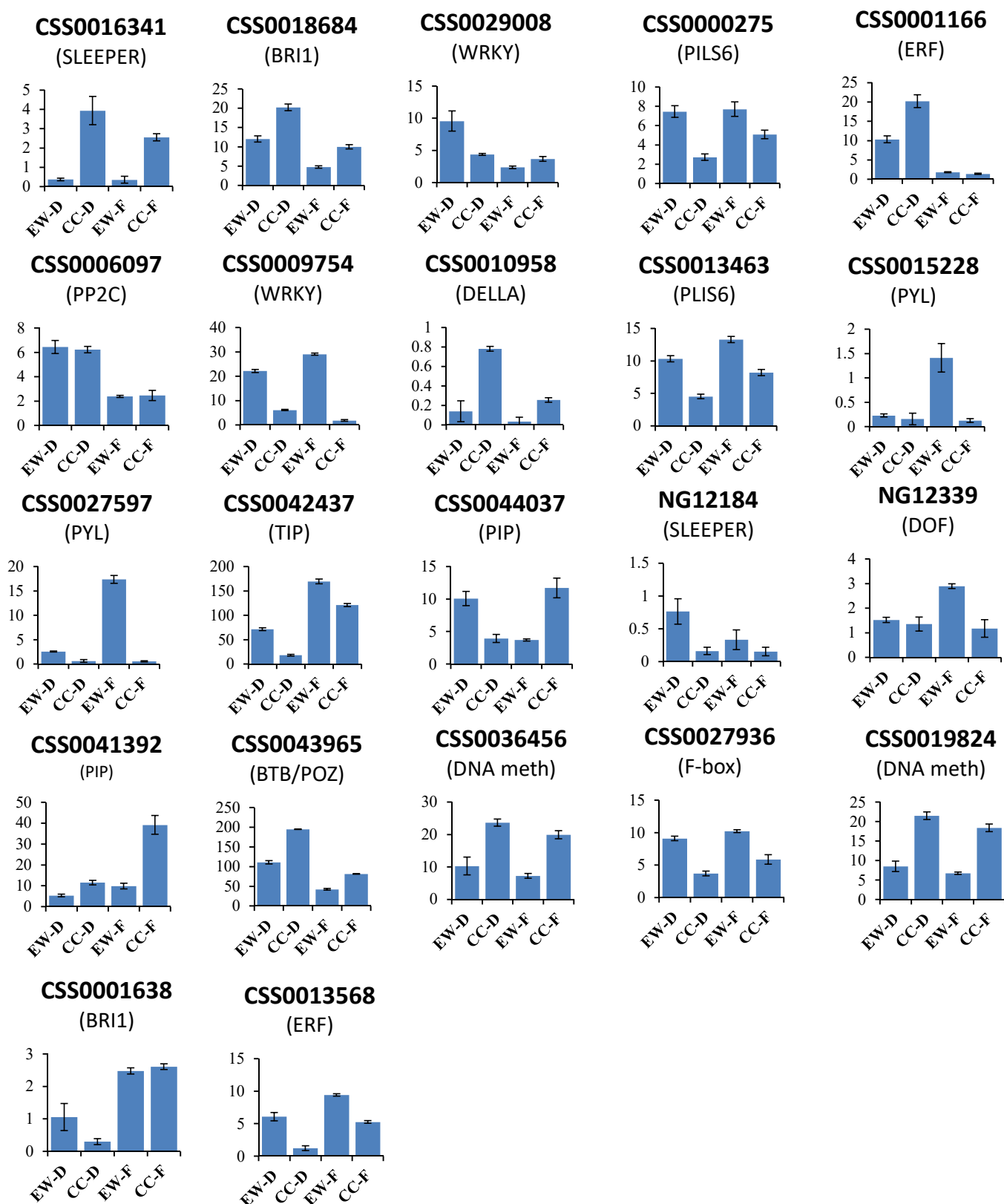

Supplement: uhac086_suppl_data [file uhac086_suppl_data.zip › Supplementary Figures.pdf]
